# Comprehensive Analysis of MILE Gene Expression Data Set Advances Discovery of Leukaemia Type and Subtype Biomarkers

**DOI:** 10.1007/s12539-017-0216-9

**Published:** 2017-03-16

**Authors:** Wojciech Labaj, Anna Papiez, Andrzej Polanski, Joanna Polanska

**Affiliations:** 1 Silesian University of Technology, Institute of Informatics, Akademicka 16, 44-100 Gliwice, Poland; 2 Silesian University of Technology, Institute of Automatic Control, Akademicka 16, 44-100 Gliwice, Poland

**Keywords:** Batch effect, Leukaemia, Biomarker identification, Gene expression, High-throughput

## Abstract

Large collections of data in studies on cancer such as leukaemia provoke the necessity of applying tailored analysis algorithms to ensure supreme information extraction. In this work, a custom-fit pipeline is demonstrated for thorough investigation of the voluminous MILE gene expression data set. Three analyses are accomplished, each for gaining a deeper understanding of the processes underlying leukaemia types and subtypes. First, the main disease groups are tested for differential expression against the healthy control as in a standard case-control study. Here, the basic knowledge on molecular mechanisms is confirmed quantitatively and by literature references. Second, pairwise comparison testing is performed for juxtaposing the main leukaemia types among each other. In this case by means of the Dice coefficient similarity measure the general relations are pointed out. Moreover, lists of candidate main leukaemia group biomarkers are proposed. Finally, with this approach being successful, the third analysis provides insight into all of the studied subtypes, followed by the emergence of four leukaemia subtype biomarkers. In addition, the class enhanced DEG signature obtained on the basis of novel pipeline processing leads to significantly better classification power of multi-class data classifiers. The developed methodology consisting of batch effect adjustment, adaptive noise and feature filtration coupled with adequate statistical testing and biomarker definition proves to be an effective approach towards knowledge discovery in high-throughput molecular biology experiments.

**Electronic supplementary material:**

The online version of this article (doi:10.1007/s12539-017-0216-9) contains supplementary material, which is available to authorized users.

## Introduction

Leukaemia as a common cancer type, nowadays still requires improvement in the domain of diagnostics and classification. Currently, modern molecular biology techniques are being assessed for their adequacy toward the detection and distinction between leukaemia subtypes. This task has been undertaken in several attempts. According to Andreeff et al. [1] in 1980 flow cytometric analysis of DNA and RNA has been used to recognize acute lymphoblastic leukaemia subtypes. Along with the creation of microarray technologies new opportunities emerged and in 1999 Golub et al. [2] discriminated between acute lymphoblastic and myeloid leukaemia (ALL, AML) types using expression data. Furthermore, gene expression profiling was used to classify paediatric ALL subtypes by Yeoh et al. [3]. Recently, apart from microarray technology for questions such as AML subtype determination [4], interest has been also turned towards searching for leukaemia biomarkers with miRNA [5–10] and lncRNA [11] analysis. Despite the existence of all those studies, there remains only one exceptional study which was conducted on a large scale to discriminate between all of the leukaemia subtypes [12].

This data set has been established with a great understanding of the importance of experimental design methods developed within the scientific community nowadays. The principles of control, replication and randomisation are commonly known and implemented throughout laboratories and research institutions regardless of the study field. This enables planning of complex experiments, such as the Microarray Innovations in leukaemia (MILE) [12]. It has been carried out with comprehensive state-of-the-art protocols and strict control procedures during the experimental stage. This was expected to lead to higher power of statistical testing, and thus a better chance of obtaining meaningful novel results. Still, the rich data set offers possibilities for further conclusions if deeper attention is directed towards the preprocessing and downstream analysis pipelines.

On the basis of this study, devoted to biomarker discovery, the presented work has the objective of demonstrating how considerate and custom data preprocessing is essential to the inference by reducing the chance of false discoveries. It has a substantial impact on the final conclusions, which proves how it should be commonly unthinkable to neglect this indispensable step in biomedical data mining.

## Materials and Methods

### Data Sets

The Microarray Innovations in Leukaemia (MILE) study [12] was designed to assess the clinical accuracy of gene expression profiles, originating from microarray experiments, compared to standard leukaemia laboratory methods (*gold standard*) for 16 acute and chronic leukaemia subclasses, myelodysplastic syndromes (MDSs) and control group that included non-malignant disorders and normal bone marrow. The leukaemia subclasses may be divided into four main groups: acute and chronic myeloid leukaemia (AML, CML) and acute and chronic lymphoblastic leukaemia (ALL, CLL). The investigation was performed in 11 laboratories across three continents and included a total of 3,334 patients. The study was very carefully designed to eliminate main problems, which occur when many experiments are carried out in various laboratories in diverse conditions—so called *batch effect* [13]. The experiments consisted of four phases: two main phases (Stage I and Stage II), each of them preceded by a pre-phase [14]. The goals of the pre-phases were to assure intra laboratory reproducibility and inter laboratory comparability. Each laboratory operator was trained on an identical sample preparation protocol. Additionally, each laboratory was provided with the same laboratory equipment and also kits and reagents for sample preparation and microarray analysis were taken from the same source.

In this analysis microarray data from Stage I of the MILE study were investigated, where 2096 bone marrow samples of acute and chronic leukaemia patients were hybridized to Affymetrix HG-U133 Plus 2.0 GeneChips. Summary of the MILE datasets Stage I is presented in Table 1.

**Table 1:** Summary of the MILE datasets (STAGE I)

Class	Diagnosis	No of samples
B-ALLt(8;14)	Mature B-ALL with t(8;14)	13
Pro-B-ALLt(11q23)	Pro-B-ALL with t(11q23)/MLL	70
Pre-B-ALLt(9;22)+	c-ALL/pre-B-ALL with t(9;22)	122
T-ALL	T-ALL	174
ALLt(12;21)	ALL with t(12;21)	58
ALLt(1;19)	ALL with t(1;19)	36
ALLhk	ALL with hyperdiploid karyotype	40
Pre-B-ALLt(9;22)−	c-ALL/pre-B-ALL without t(9;22)	237
AMLt(8;21)	AML with t(8;21)	40
AMLt(15;17)	AML with t(15;17)	37
AMLt(16;16)	AML with inv(16)/t(16;16)	28
AMLt(11q23)	AML with t(11q23)/MLL	38
AMLnk	AML with normal karyotype + other abnormalities	351
AMLcak	AML complex aberrant karyotype	48
CLL	CLL	448
CML	CML	76
MDS	MDS	206
CTR	Non-leukaemia and healthy bone marrow	74
Total		2096

Types of leukaemia defined by gold standard methods in the experimental protocol

Three comparison studies were accomplished following the same signal analysis pipeline. Two of three analyses were performed on main classes of leukaemia and, therefore, merging samples from the appropriate subclasses was needed. The summary of merged data is presented in Table 2.

**Table 2:** Samples in main classes of leukaemia after subclass merging using the MILE datasets in STAGE I

Diagnosis	Main classes	No of samples	Diagnosis	Main classes	No of samples
B-ALLt(8;14)	ALL	750	AMLt(8;21)	AML	542
Pro-B-ALLt(11q23)			AMLt(15;17)		
Pre-B-ALLt(9;22)+			AMLt(16;16)		
T-ALL			AMLt(11q23)		
ALLt(12;21)			AMLnk		
ALLt(1;19)			AMLcak		
ALLhk			CLL	CLL	448
Pre-B-ALLt(9;22)−			CML	CML	76
MDS	MDS	206	CTR	CTR	74
Total					2096

### Analysis Pipeline

**Fig. 1:**
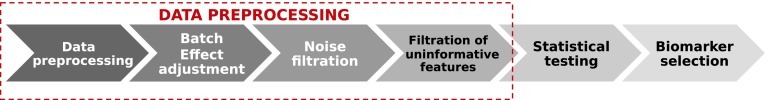
Summary of pipeline analysis, which includes preprocessing data, batch effect adjustment, adaptive filtering for noise and uninformative feature filtration, statistical analysis and biomarker selection

Taking into account the specific nature of the data set, the pipeline of analysis was designed as presented in Fig 1. It includes the use of state of the art methods for preprocessing, a technique for removing variability caused by external influence (unrelated to the analysed case), adaptive filtering for noise and uninformative features removal, statistical analysis with the aim of biomarker selection.

The three comparative analyses performed gradually take into account more and more details about the leukaemia. The first one is carried out on the main types of leukaemia, and in terms of statistical analysis a commonly used approach is chosen, which compares the mean gene expression level in each main type of leukaemia with the mean expression level among healthy donors from control group (here: non-leukaemia and healthy bone marrow). This is an example of case-control approach widely used in observational studies.

In the second analysis, an extension is performed relying on the cross-comparison of transcriptomic profiles of main leukaemia types between themselves. From this analysis a biomarker identification step is added as it is possible to set an appropriate condition. In this case only such features are taken into account and labelled as biomarkers, which differentiate one and only one main class from the rest.

The last analysis was performed on all of the leukaemia subgroups. It allows for the most profound analysis of leukaemia diseases. As mentioned earlier, this is a unique study, which was conducted on a large scale to discriminate between all of the leukaemia subtypes and in this final study information for all subclasses of leukaemia is taken under analysis.

### Data Preprocessing

The intensity data from microarray experiments has been subjected to fRMA normalisation [15] with background correction, quantile normalisation and median polish summarisation. This method has been chosen to merge the advantages of classic RMA normalisation with the ability to include additional samples if need in the future. Probe reannotation was accomplished with custom CDF files available through the BrainArray repository [16].

**Fig. 2:**
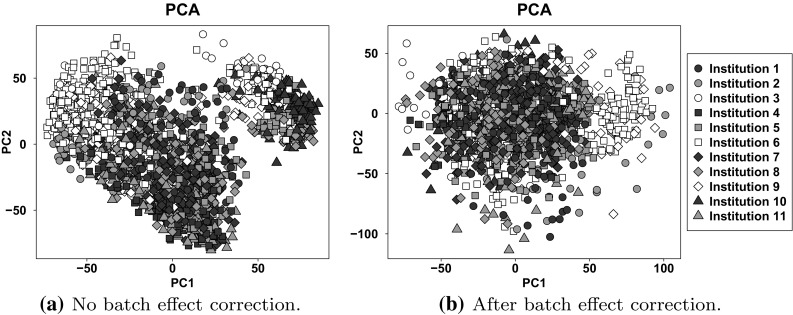
Principal component graphs demonstrating the existence of batch effect in the data with regard to sample preparation research centre

The next step was to ensure data coherence, i.e. verify if the unification procedures applied in the study successfully dealt with the issue of bias introduced by batch effect. In this case Principal Component Analysis was performed and the outcome suggests that nonetheless a batch effect due to sample preparation in different laboratories may be observed (Fig. 2).

Therefore, the data were adjusted for batch effects with the use of ComBat algorithm [17], available through the SVA R package [18]. The results of Kruskall-Wallis test for differentially expressed genes among research centre batches proved a significant removal of batch effect (Table 3).

**Table 3:** Results of two-way ANOVA for gene differentiation among research centres participating in sample preparation and leukaemia subgroups ($\alpha = 0.05$)

Total	No of genes		
	Research centres	Leukaemia subtype	Interaction
	18,988		
No batch effect correction	13,698	14,860	12,738
After batch effect correction	8	11,312	10,753

**Fig. 3:**
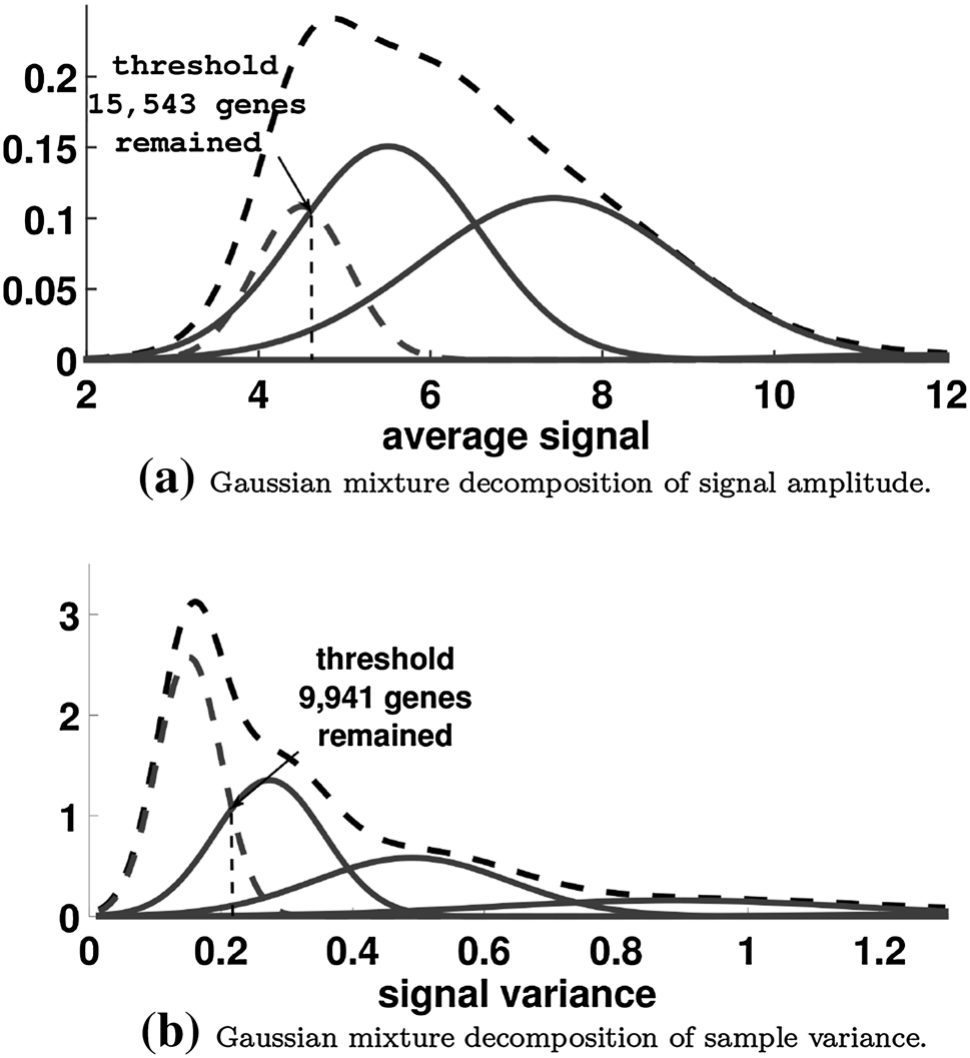
Decomposition into Gaussian components as a method of filtration of genes with signal intensity close to background values and low variance

The final step consisted of gene filtration to remove features with signal close to background level. There are various techniques available for this purpose such as the commonly used method of removing $50\%$ of the genes with lowest expression value or variance. However, in the studied case of 18 subtypes of disease this approach seems excessively strict and implies the search of an adaptive threshold rather than fixed. For this reason, the adaptive filtering based on Gaussian mixture decomposition has been selected [19]. The filtration was conducted in two steps: in the first step the signal was decomposed in terms of signal intensity amplitude, and the three components with the highest signal amplitude remained. Second, the data were considered variance-wise and the component with lowest variance was rejected (Fig. 3). A total of 9941 genes remained for further statistical analysis.

### Statistical Analysis and Biomarker Selection

To search for class enhanced differentially expressed genes (CE-DEGs) across types or subtypes of leukaemia, a set of statistical tests was carried out, independently for each comparative analysis. The CE-DEGs in this case are genes which differentiate a considered group from all the other groups in the manner of pairwise comparisons. At the beginning the conditions on normality and homogeneity of variances were verified and, accordingly, the appropriate parametric or non-parametric test was chosen.

During the first analysis, initially, Analysis of Variance (ANOVA) was conducted to filter out the genes, which do not differentiate among groups at all. Next, the mean gene expression level of each main type of leukemia was compared with the mean expression within reference group, therefore, Dunnetts test was used in post hoc comparisons to control the experimental event rate (EER).

For the remaining two analyses the same set of statistical tests was performed. It included non-parametric Kruskal-Wallis analysis of variance test, because of the violation of the assumptions for parametric ANOVA in several experimental groups. After this step features, which differentiate at least one leukaemia type from the rest types of diseases, were selected. Furthermore, as means of conducting post-hoc pairwise comparison tests, the Games-Howell method was chosen. Restrictive feature selection was then used to filter out the genes which differentiate solely one group from all of the other types or subtypes of leukaemia. The combination of the data preprocessing steps and statistically supported biomarker selection method form an innovative pipeline for comprehensive expression data analysis.

### Cross Validation

With respect to the works presented in  [12] a similar cross validation scheme was executed for data processed in the original study and data from the proposed preprocessing and statistical testing analysis pipeline. Namely, 30-fold cross validation with three repetitions was carried out on the leukaemia subgroups using a Support Vector Machine (SVM) classifier. As a common practice to account for regularisation, the minimum error rate criterion was used in the differentiating feature selection process. Moreover, separability was measured using SVM on the entire data set for original data and processed with the proposed pipeline. The former feature set consisted of the union of top 100 differentially expressed genes from *t* test pairwise comparisons, whereas in the latter case the total number of CE-DEGs identified in the Games-Howell post-hoc test. The feature selection step was completed with the condition that genes which are incorporated into the model cannot be correlated in the sense of a large effect size value.

## Results

### Case–Control Approach: Leukaemia Versus Healthy Controls

**Fig. 4:**
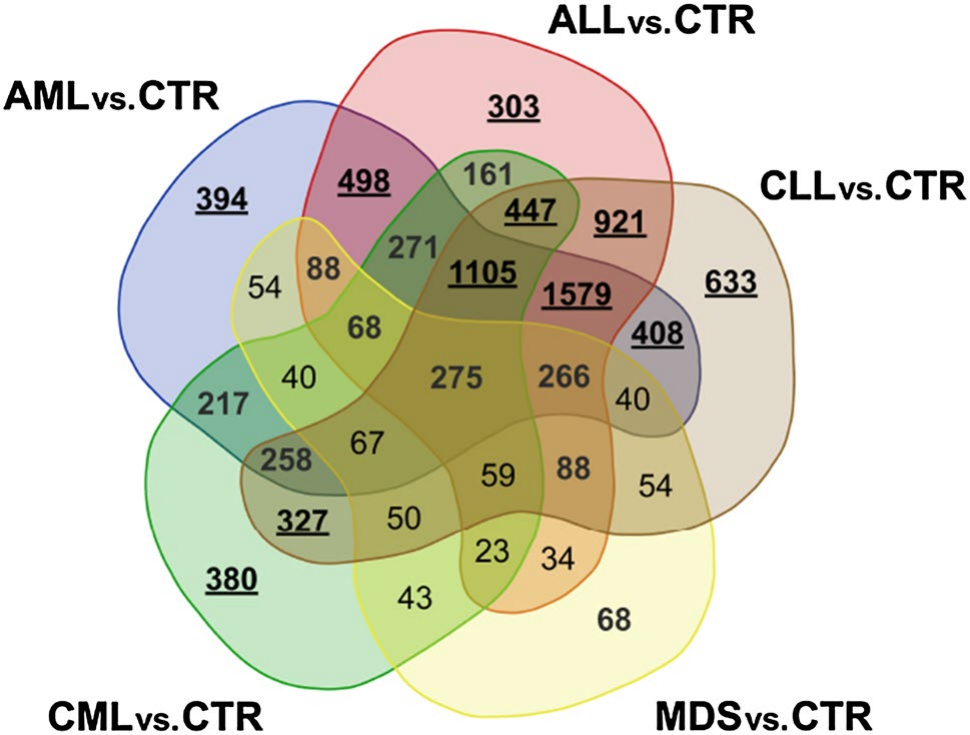
Venn diagram for differentially expressed genes in the main leukaemia subgroups with regard to their control

**Fig. 5:**
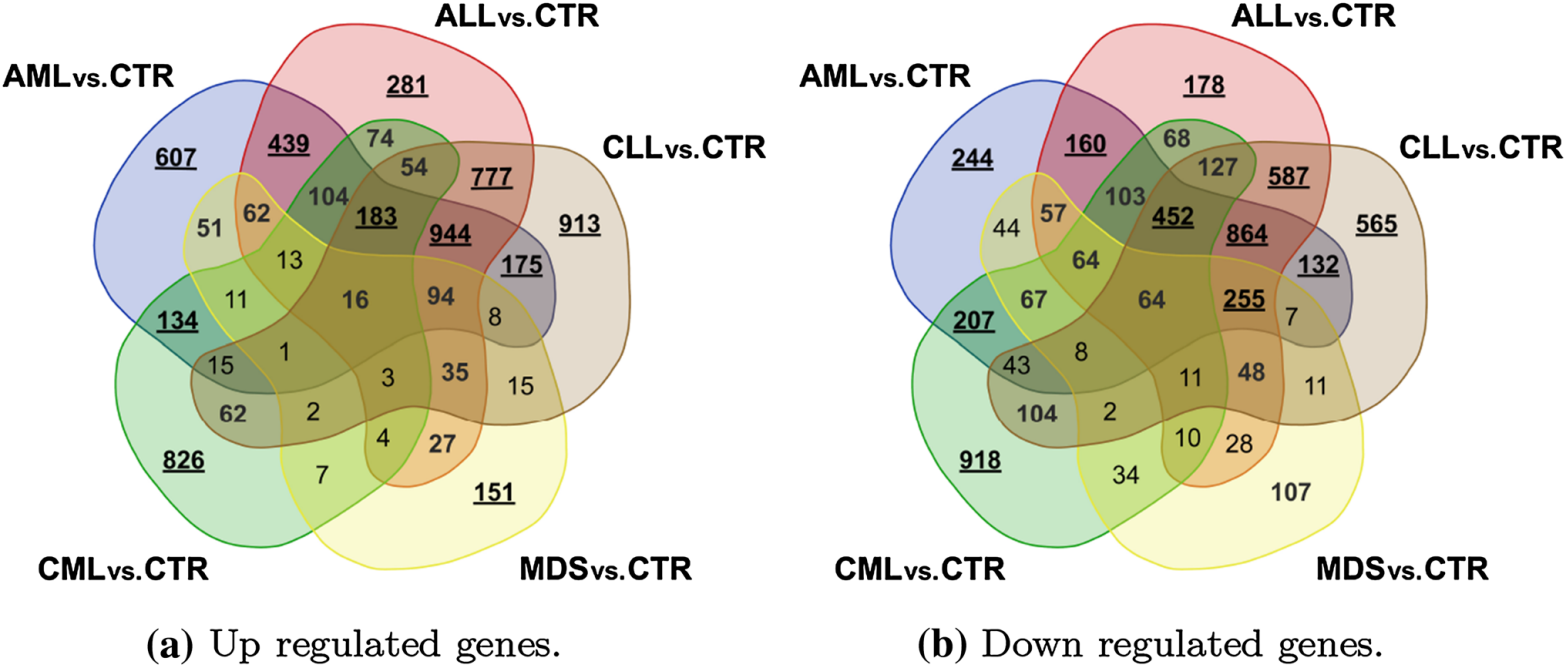
Venn diagrams for up and down regulated genes in the main leukaemia subgroups with regard to their control

The first analysis consisted of a common approach of examining differentiation between gene expression profile in samples collected from patients diagnosed with one of the main leukaemia groups and the control group. In this case the control samples are treated somewhat as a baseline and the insight is being driven towards up and down regulated genes. The summary of these findings is presented in Figs. 4 and  5. The Venn diagrams (http://bioinformatics.psb.ugent.be/webtools/Venn) present similarity among the four main leukaemia groups in terms of the sets of differentiating genes in total and taking into account the division of up and down regulated. The total number of genes differentially expressed between leukaemia and controls per each leukaemia type is presented in Table 4. As expected, the lowest number of CE-DEGs is observed for MDS cases, while ALLs, AMLs and CLLs present the similar number of CE-DEGs. There are no significant differences in the number of up and down regulated genes for ALL, AML, and CLL leukaemia type (50.27, 50.76, and 50.13$\%$ of up regulated genes), while for CML type down regulated genes overdominate the system response ($60.20\%$). A similar trend is observed for MDS samples. The complete list of differentially expressed genes with regard to the healthy controls is given in Supplementary File 1 .

The CE-DEGs have been verified through literature research for the presence of key genes present in molecular mechanisms of the studied leukaemia types. In all of the investigated diseases these principal features appeared to be significantly altered in terms of gene expression. Hence, the lists of CE-DEGs included:

- ALL: EBF1, LMO2, CDKN2A, PTEN, RB1, BTLA, CD200, TOX, NR3C1, TBL1XR1, ETV6, ERG genes reported to be linked with acute lymphoblastic leukaemia [20, 21]
- AML: FLT3, IDH1, DNMT3A, CEBPA, KIT, NRAS, NPM1 genes connected with acute myeloid leukaemia [22]
- CLL: ATM, GPI, BSG, LGALS1, PARVB, VIM, NOTCH1, BIRC3, MYD88, CD38 associated with chronic lymphoblastic leukaemia as in [23, 24]
- CML: has been confirmed to have, among others, a significantly differentially expressed BCR-ABL gene, which is the leading oncoprotein involved in chronic myeloid leukemia [25, 26]

**Table 4:** The number of statistically significant differentiating genes for each of the main groups of leukaemia with regard to the control sample group

Leukaemia type	ALL	AML	CLL	CML	MDS
No of differentiating genes	6186	5628	6577	3791	1317
No of up regulated genes	3110	2857	3297	1509	500
No of down regulated genes	3076	2771	3280	2282	817

The similarity has been further determined by means of the Dice coefficient [27] (DSC) with its 95% confidence intervals [28] (Table 5). These statistics show that the most substantial resemblance is within the genes differentially expressed in ALL and CLL, although a powerful similarity is also present between the AML and ALL groups. The least important closeness may be seen in the case of each main leukaemia group when compared to MDS. Detailed analysis of DSC values between MDS and leukaemia types reveals that MDS is the most similar to AML in systemic response to disease, having significantly the highest value of Dice similarity coefficients (0.259; $95\%$ CI from 0.245 to 0.272), which is in compliance with the findings of other authors [29].

**Table 5:** Dice coefficients (DSC) with confidence intervals for main groups of leukaemia comparison

	ALL	ALL	ALL	ALL	AML	AML	AML	CLL	CLL	CML
	&	&	&	&	&	&	&	&	&	
	AML	CLL	CML	MDS	CLL	CML	MDS	CML	MDS	MDS
CE-DEGs										
−95%CI	0.693	0.734	0.471	0.228	0.645	0.476	0.245	0.487	0.216	0.229
DSC	**0.703**	**0.743**	**0.483**	**0.240**	**0.655**	**0.489**	**0.259**	**0.499**	**0.228**	**0.245**
+95%CI	0.712	0.751	0.495	0.253	0.665	0.501	0.272	0.511	0.240	0.261
Down regulated CE-DEGs										
−95%CI	0.677	0.746	0.319	0.258	0.589	0.382	0.296	0.276	0.182	0.150
DSC	**0.691**	**0.758**	**0.336**	**0.276**	**0.603**	**0.399**	**0.316**	**0.292**	**0.198**	**0.168**
−95%CI	0.704	0.769	0.352	0.295	0.618	0.416	0.335	0.307	0.215	0.186
Up regulated CE-DEGs										
−95%CI	0.607	0.644	0.180	0.126	0.451	0.202	0.136	0.127	0.079	0.044
DSC	**0.622**	**0.657**	**0.195**	**0.141**	**0.467**	**0.219**	**0.153**	**0.140**	**0.092**	**0.057**
−95%CI	0.636	0.671	0.211	0.157	0.482	0.235	0.169	0.154	0.105	0.072

The rows present similarity measures for the total number of CE-DEGs, as the lower CI bound, Dice coefficient (in bold) and upper CI bound for and with the distinction between up and down regulated genes

### Comparison Among Leukaemia Main Types

**Fig. 6:**
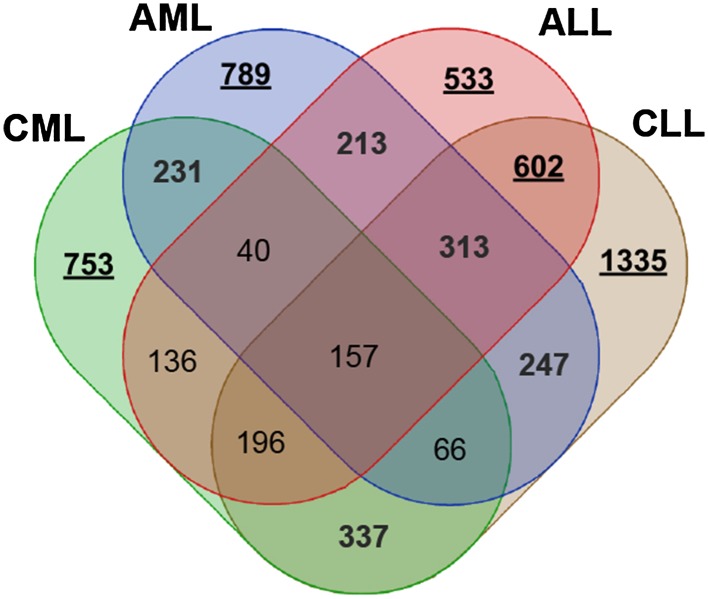
Overlap of class enhanced differentially expressed genes among main leukaemia groups

In the second analysis, the main leukaemia types have been investigated using pairwise comparison testing to identify possible biomarkers among main groups of leukaemia. In this case, apart from being differentially expressed, the gene had to be uniquely statistically significant for only the one leukaemia type in order to be recognized as a potential biomarker (in contrast to CE-DEGs, which could differentiate several groups from each other). It cannot be differentially expressed among remaining leukaemia types. The findings have been summarised in Table 6 and on Fig. 6, while the complete lists of genes are available in Supplementary File 2.

**Table 6:** Results of Games-Howell post-hoc pairwise comparisons

Leukaemia type	ALL	AML	CLL	CML	MDS	CTR
No of CE differentiating genes	2190	2056	3253	1916	509	357
Leukaemia type biomarkers	42	55	68	58	2	3

The type biomarkers are genes which differentiate only a particular type of disease from all other classes

Thereafter, the biomarkers were subjected to functional analysis for gaining knowledge of biological processes, in which they may be involved. Therefore, they have been checked for links to biological process terms in the Gene Ontology database [30]. The biomarker lists were submitted for Gene Ontology overrepresentation assessment using Fishers exact test. Nearly complete dissimilarity of the discovered overrepresented GO terms points to an apparent specificity of biological processes triggered by genes differentially expressed in the forenamed leukaemia types. The complete lists of ontology terms are gathered in Supplementary File 3.

Moreover, the biomarker genes have been juxtaposed with regard to their gene family for a more complete information set on the connection between their function and potential leukaemia-related processes. INGENUITY^TM^ Pathway Analysis software by QIAGEN was used for this purpose. The summary of the outcome is presented in Table 7. The findings point to a few notable indications, i.e. the presence of growth factors only in acute leukaemia and phosphatases in myeloid leukaemia. Furthermore, the occurrence of the G-protein coupled receptor family is specific for ALL, peptidase for AML, transmembrane receptors for CLL and microRNA for CML.

**Table 7:** List of gene family characteristics for main leukaemia type candidate biomarkers

Gene family	ALL	AML	CLL	CML
Enzyme	8	10	13	11
G-Protein coupled receptor	1	0	0	0
Growth factor	2	1	0	0
Transcription regulator	6	3	0	1
Cytokine	1	0	0	1
Ion channel	2	1	1	1
Transporter	2	6	6	0
Kinase	0	4	5	2
Peptidase	0	1	0	0
Phosphatase	0	1	0	3
Transmembrane receptor	0	0	2	0
MicroRNA	0	0	0	1
Other	20	28	41	38

### Searching for Leukaemia Subtype Biomarkers

**Fig. 7:**
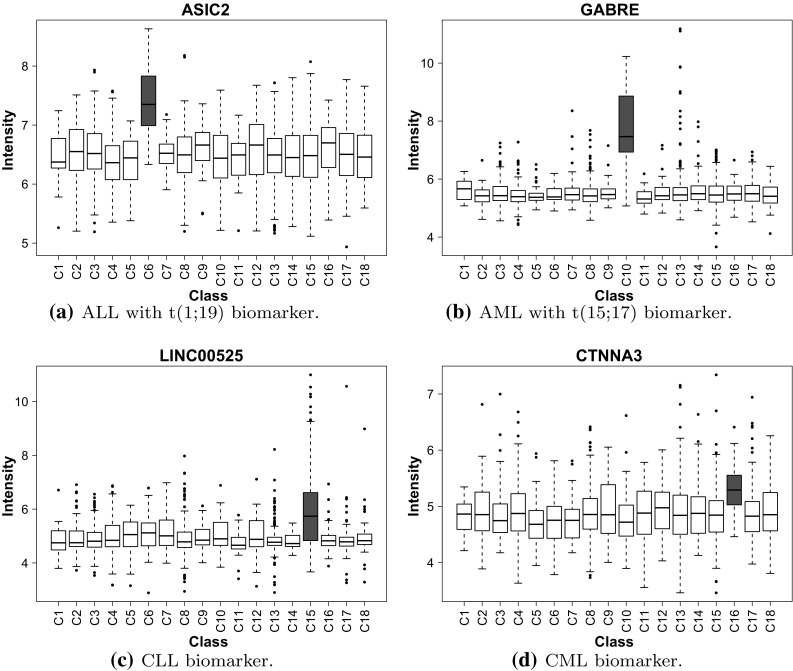
Boxplots illustrating the biomarker gene expression value distributions for subclasses of leukaemia

Having the required measurements for gaining insight into the individual leukaemia subgroups, the data were investigated in a deeper manner and the analysis pipeline (Fig. 1) was repeated for all of the eighteen leukemia subtypes. Differentiation testing results demonstrate that an overwhelming majority of the genes remaining for analysis present statistical significance between the studied subgroups of leukemia (Table 3). After adequate gene filtration it is highly probable that at least one type will vary from the others significantly. Thus, pairwise comparisons were carried out between the subgroups and the final results (Table 8) pointed out to merely four genes differentiating a subgroup from all the others. The genes mentioned are (Fig. 7): (1) ASIC2 acid sensing ion channel 2, (2) GABRE—gamma-aminobutyric acid A receptor, epsilon, (3) LINC00525—long intergenic non-protein coding RNA 525, (4) CTNNA3—catenin alpha 3. The CTNNA3 gene has been shown to be linked to the Shwachman-Diamond syndrome which is characterized by a high risk of leukaemia [31]. In terms of relation to the bone marrow processes the GABRE gene which is a gammaaminobutyric acid receptor has proved to play a role during bone marrow stromal cell transplantation in the injured spinal cord in mice [32].

**Table 8:** Results of Games-Howell post-hoc pairwise comparisons

Leukaemia subtype	B-ALL	Pro-B-ALL	Pre-B-ALL	T-ALL	ALL	ALL
	t(8;14)	*t*(11q23)	*t*(9;22)+		*t*(12;21)	*t*(1;19)
No of CE-DEG	11	46	18	111	136	90
Subtype biomarkers	0	0	0	0	0	1

Leukaemia subtype	ALLhk	Pre-B-ALL	AML	AML	AML	AML
		*t*(9;22)	*t*(8;21)-	*t*(15;17)	*t*(16;16)	*t*(11q23)
No of CE-DEG	105	2	27	141	36	19
Subtype biomarkers	0	0	0	1	0	0

Leukaemia subtype	AMLnk	AMLcak	CLL	CML	MDS	CTR
No of CE-DEG	4	3	18	90	1	2
Subtype biomarkers	0	0	1	1	0	0

The subtype biomarkers are genes which differentiate only a particular subtype of disease from all the other subclasses

### Classification Study

The cross validation results in detail are presented in Tables 9 and 10. The prediction for all of the leukaemia subclasses is given along with classification sensitivity. Furthermore, overall weighted average sensitivity with $95\%$ confidence intervals is presented in Table 11. It is visible that features selected through the proposed analysis pipeline have higher average specificity than those chosen with the top 100 DEG original approach.

In terms of separability, there were 39 genes in the model for data processed with the original approach and 41 in the novel pipeline approach. Two of these features were common and the remaining were correlated with effect size at least at a medium level. the results are on a similar level. The identified novel pipeline signature is driven by leukaemia known MEIS1, CBFB, FOXO1, SETBP1 genes with the support, among the others, of KIAA0101, GPX1, INSR HCCS and THOC5 genes. The complete list of genes in the signature is available in Supplementary File 4. The majority of them has been previously reported to be linked to leukaemia related processes. Using the novel pipeline 0.998 accuracy was reached with the minimum error rule, versus 0.972 for the original MILE approach. However, less iterations for the procedure were required in case of the novel pipeline, as the considered feature space was smaller (2316 CE-DEGs) than in the original approach (3555 genes).

**Table 9:** Prediction table with cross validation results for the original MILE analysis pipeline data for leukaemia subgroups

Class	C1	C2	C3	C4	C5	C6	C7	C8	C9	C10	C11	C12	C13	C14	C15	C16	C17	C18	Total	Sensitivity
MILE cross validation																				
C1	–	–	–	1.0	–	–	–	–	–	–	–	–	8.0	–	2.0	–	2.0	–	13	0.000
C2	–	54.3	12.0	–	–	–	–	–	–	–	–	–	3.7	–	–	–	–	–	70	0.776
C3	–	1.0	203.3	3.0	4.3	–	2.3	13.0	–	–	–	–	5.3	–	1.3	–	2.0	1.3	237	0.858
C4	–	–	1.0	151.3	–	–	–	–	–	–	–	–	18.0	–	1.0	–	1.7	1.0	174	0.870
C5	–	–	26.7	–	30.3	–	–	–	–	–	–	–	–	–	–	–	–	1.0	58	0.523
C6	–	1.0	26.7	–	–	7.7	–	–	–	–	–	–	0.7	–	–	–	–	–	36	0.213
C7	–	–	29.0	–	1.0	–	8.7	0.3	–	–	–	–	1.0	–	–	–	–	–	40	0.217
C8	–	–	58.7	1.0	–	–	1.0	51.0	–	–	–	–	8.7	–	1.0	–	0.3	0.3	122	0.418
C9	–	–	–	–	–	–	–	–	17.7	–	–	–	18.7	–	–	3.0	0.7	–	40	0.442
C10	–	–	–	–	–	–	–	–	–	24.7	–	–	11.0	–	–	–	1.3	–	37	0.667
C11	–	–	–	–	–	–	–	–	–	–	6.7	–	21.3	–	–	–	–	–	28	0.238
C12	–	–	–	0.3	–	–	–	–	–	–	–	–	36.7	–	–	1.0	–	–	38	0.000
C13	–	–	4.0	5.3	–	–	–	1.0	–	–	0.7	–	312.3	–	2.0	4.0	21.7	–	351	0.890
C14	–	–	–	–	–	–	–	–	–	–	–	–	39.0	–	1.0	–	8.0	–	48	0.000
C15	–	–	–	1.0	–	–	–	–	–	–	–	–	8.3	–	438.7	–	–	–	448	0.979
C16	–	–	1.0	–	–	–	–	–	–	–	–	–	7.3	–	–	57.0	10.7	–	76	0.750
C17	–	–	–	–	–	–	–	–	–	–	–	–	26.0	–	2.3	1.0	173.3	3.3	206	0.841
C18	–	–	–	–	–	–	–	–	–	–	–	–	3.0	–	1.0	1.0	56.7	12.3	74	0.167

**Table 10:** Prediction table with cross validation results for the novel proposed analysis pipeline data for leukaemia subgroups

Class	C1	C2	C3	C4	C5	C6	C7	C8	C9	C10	C11	C12	C13	C14	C15	C16	C17	C18	Total	Sensitivity
Novel approach cross validation																				
C1	3.0	–	1.0	–	–	–	–	–	–	–	–	–	2.0	–	4.0	–	2.0	1.0	13	0.231
C2	–	68.0	1.0	–	–	–	–	–	–	–	–	–	–	–	1.0	–	–	–	70	0.971
C3	–	2.0	189.0	2.0	–1.0	–	15.0	5.0	–	–	–	–	2.3	0.3	3.7	–	7.0	0.7	237	0.797
C4	–	–	–	155.7	–	–	–	–	–	–	–	–	10.3	–	5.0	1.0	2.0	–	174	0.895
C5	–	–	2.3	–	53.7	–	–	–	–	–	–	–	–	–	1.0	–	–	1.0	58	0.926
C6	–	2.0	1.7	–	–	31.0	–	–	–	–	–	–	–	–	1.3	–	–	–	36	0.861
C7	–	–	9.7	–	–	–	30.3	–	–	–	–	–	–	–	–	–	–	–	40	0.758
C8	–	–	12.3	–	–	–	1.0	101.3	–	–	–	–	4.0	–	2.0	1.0	–	0.3	122	0.830
C9	–	–	–	–	–	–	–	–	37.3	–	–	–	1.7	–	1.0	–	–	–	40	0.933
C10	–	–	–	–	–	–	–	–	–	32.0	–	–	4.0	–	–	–	1.0	–	37	0.865
C11	–	–	–	–	–	–	–	–	–	–	28.0	–	–	–	–	–	–	–	28	1.000
C12	–	–	–	1.0	–	–	–	–	–	–	–	$-2.0$	15.0	–	1.0	1.0	–	–	38	0.526
C13	1.0	–	1.0	3.0	–	–	–	–	–	1.0	–	1.0	315.7	–	7.3	1.0	$-2.0$	–	351	0.899
C14	–	–	–	–	–	–	–	–	–	–	–	–	$-3.0$	9.0	5.0	–	4.0	–	48	0.188
C15	–	–	–	–	–	–	–	–	–	–	–	–	2.3	–	445.0	–	0.7	–	448	0.993
C16	–	–	1.0	–	–	–	–	–	–	–	–	–	1.0	–	2.3	67.3	4.3	–	76	0.886
C17	–	–	–	–	–	–	–	–	–	–	–	–	19.7	–	1.7	1.0	178.3	5.3	206	0.866
C18	–	–	–	–	–	–	–	–	–	–	–	–	–	–	2.0	1.0	31.3	39.7	74	0.536

**Table 11:** Weighted average cross validation sensitivity with $95\%$ CI for the original MILE data and the novel processing pipeline

	Specificity	95% Confidence interval
Original MILE approach	0.739	(0.737;0.741)
Novel analysis pipeline	0.861	(0.860;0.862)

## Discussion

The analysed data originate from one of the main phases of MILE study and contain 2096 samples prepared by 11 research centres from around the world. This may be the cause of impairment of the quality of data by the impact of technical factors related to each research centre. However, the whole experiment was very well designed, which means every laboratory was provided with the same equipment, kits, reagents coming from a common manufacturer or source. Likewise, the technicians were prepared in terms of using identical sample preparation protocol. As a result, the data should not have been greatly affected by bias.

The analysis adapted to the specific nature of the analysed data revealed that despite a well designed experiment, variability exists in the data associated with sample preparation by particular research institutes. This prompted batch effect adjustment of which the effects are presented both in the illustration of PCA components and also by analysis of variance using two-way ANOVA for research institutions, before and after batch effect correction. The presented study indicates that batch effect correction should be an indispensable element of the microarray analysis protocol, as often it is impossible to exclude the impact of all external factors.

The three comparative immersing analyses provide advancing knowledge on the potential mechanisms of particular leukaemia types and subtypes. The first one supports findings such as an important similarity of changes in gene expression between the same tissue type (ALL and CLL). Moreover, the acute leukaemia types (AML and ALL) also appear to have multiple shared molecular responses given their number of common CE differentially expressed genes. Additionally, the MDS studied subtype seems to have the least similar gene expression set with regard to the main leukaemia subtypes, of which AML was the mostly targeted by the same genes. This, together with a relatively small number of CE-DEGs in total, may point toward the suggestion that MDS is in its mechanisms more related to a healthy response than to any of the leukaemia types.

The second main leukaemia type comparative analysis supplies further evidence toward the similarity of ALL vs. CLL and AML vs. ALL gene expression wise. The abundance of differentiating features lead to the formulation of a biomarker definition such that only genes significant for a unique type are considered candidates. This implied a reduction in number of examined genes and investigating corresponding overrepresented gene ontology terms guides more towards a conclusion that a majority of the biological processes involved in leukaemia are specific to the aforementioned main types. Furthermore, the investigation of gene families presents some guidance toward inferring that while there are gene types specific for each of the main leukaemia groups, growth factors seem to be a linking factor for acute leukaemia, whereas phosphatases for myeloid leukaemia.

The final study involving deep data analysis of all of the subtypes of leukaemia allowed the extraction of important information. Four genes were discovered (ASIC2, GABRE, LINC00525, CTNNA3) as candidate biomarkers for four subtypes of leukaemia (ALL with t(1;19), AML with t(15;17), CML, CLL). Two of them are already described in the literature. Information, which has been found in the course of literature research, coincides to some extent with information about CTNNA3 and GABRE gene involvement in branches of diseases associated with leukaemia. However, the discovered ASIC2 and LINC00525 biomarkers are not mentioned in the literature in this context and would require experimental confirmation to contribute final proof for the utility of these biomarkers.

Cross validation comparison of the original approach versus tailored preprocessing and statistical testing reveal that adequate gene set selection yields supreme results in terms of classification sensitivity. Additionally, comparative separability assessment demonstrates that with a similar level of separability is possible to obtain with a smaller gene set, which, apart from reducing the chance of finding false positives, diminishes the number of iterations that need to be performed in a classification scheme. This may be considered as significant in terms of computational resources necessary for performing analyses.

## Conclusions

The presented research confirms the significance of careful data preprocessing including batch effect adjustment and adaptive filtration for inference in a well designed large study of gene expression data in leukaemia patients. The above has been confirmed through statistical and functional analysis supported by bioinformatics repository information and literature survey of the biological conclusions. The obtained outcome produced four candidate biomarkers which imply further investigation through data mining procedures. The unique candidate biomarkers that have not been previously described in literature require experimental assessment to ultimately validate their suitability as auxiliary indicators of disease subtypes in leukaemia.

The contribution of the study is the original design of the data analysis pipeline tailored to large, multiclass, bioinformatic data. Compared to standard techniques the proposed design includes two-fold modifications. The first modification is in the preprocessing stage, more careful and elaborate, which allows for better reducing of measurement artifacts in the data while keeping the useful information. The second modification is the procedure for choice of the differentially expressed genes. We point out that in the multiclass experiments the concept of DEGs becomes more complex than in the two-class case. We introduce the definition of the class enhanced DEG and biomarkers. CE-DEG is a feature, which shows differential expression between the given class and all remaining classes grouped together. A biomarker is a CE-DEG, which additionally has a property that it does not show differential expression between any pair of the remaining classes. We apply the proposed data analysis pipeline to the MILE dataset and we demonstrate that the list of the obtained CE-DEGs, while comparable in size, is different than the list of DEGs computed in the MILE study. We also prove that our CE-DEG signature leads to significantly better classification power of the multi-class data classifiers.

**Fig. 8:**
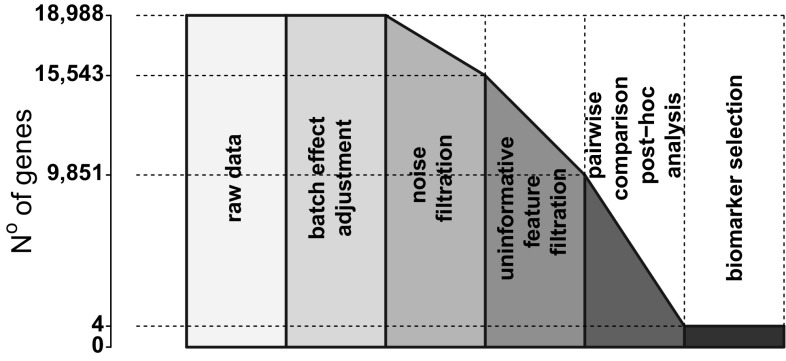
Expression data analysis pipeline with gradual gene reduction

In conclusion, the provided deep data analysis pipeline (Fig. 8) proves to be an advantageous tool for screening high-throughput molecular biology data sets.

## Electronic supplementary material

Below is the link to the electronic supplementary material.

Supplementary material 1 (XLSX 561 kb)

Supplementary material 2 (XLSX 15 kb)

Supplementary material 3 (XLSX 60 kb)

Supplementary material 4 (XLSX 7 kb)
